# Interdisciplinary eHealth Practice in Cancer Care: A Review of the Literature

**DOI:** 10.3390/ijerph14111289

**Published:** 2017-10-25

**Authors:** Anna Janssen, Melissa Brunner, Melanie Keep, Monique Hines, Srivalli Vilapakkam Nagarajan, Candice Kielly-Carroll, Sarah Dennis, Zoe McKeough, Tim Shaw

**Affiliations:** 1 Faculty of Health Sciences, The University of Sydney, Sydney, NSW 2141, Australia; melissa.brunner@sydney.edu.au (M.B.); melanie.keep@sydney.edu.au (M.K.); monique.hines@sydney.edu.au (M.H.); candice.kielly-carroll@sydney.edu.au (C.K.-C.); sarah.dennis@sydney.edu.au (S.D.); zoe.mckeough@sydney.edu.au (Z.M.); tim.shaw@sydney.edu.au (T.S.); 2 Faculty of Education and Arts, The University of Newcastle, Newcastle, NSW 2308, Australia; 3 Sydney Medical School, The University of Sydney, Sydney, NSW 2141, Australia; srivalli.nagarajan@sydney.edu.au; 4 Ingham Institute of Applied Medical Research, Liverpool, NSW 2170, Australia

**Keywords:** eHealth, interdisciplinary, cancer

## Abstract

This review aimed to identify research that described how eHealth facilitates interdisciplinary cancer care and to understand the ways in which eHealth innovations are being used in this setting. An integrative review of eHealth interventions used for interdisciplinary care for people with cancer was conducted by systematically searching research databases in March 2015, and repeated in September 2016. Searches resulted in 8531 citations, of which 140 were retrieved and scanned in full, with twenty-six studies included in the review. Analysis of data extracted from the included articles revealed five broad themes: (i) data collection and accessibility; (ii) virtual multidisciplinary teams; (iii) communication between individuals involved in the delivery of health services; (iv) communication pathways between patients and cancer care teams; and (v) health professional-led change. Use of eHealth interventions in cancer care was widespread, particularly to support interdisciplinary care. However, research has focused on development and implementation of interventions, rather than on long-term impact. Further research is warranted to explore design, evaluation, and long-term sustainability of eHealth systems and interventions in interdisciplinary cancer care. Technology evolves quickly and researchers need to provide health professionals with timely guidance on how best to respond to new technologies in the health sector.

## 1. Introduction

Globally, cancer is recognized as a significant cause of morbidity and mortality. A 2012 report estimated that there are approximately 14 million new cases of cancer diagnoses each year, a rate set to increase by 70% over the next twenty years [1]. The significant global impact of the condition has been recognized by the World Health Organization who have set a target to reduce premature mortality from cancer, alongside cardiovascular disease, diabetes and chronic respiratory disease, by 25% before 2025 [2].

In order to improve outcomes and optimize the delivery of care to patients, the health workforce has been moving towards the use of interdisciplinary approaches to care. The term interdisciplinary describes the act of collaboration between a group of professionals for a shared purpose [3]. The interdisciplinary approach is widespread in the health sector, and is frequently facilitated through the use of multidisciplinary teams (MDTs) for the delivery of care. MDTs are a sub-set of interdisciplinary care, describing a cohesive group of health professionals who work together on a regular basis. The literature indicates that the primary function of MDTs is to bring together health professionals from multiple disciplines, including radiology, surgery, nursing and allied health, to deliver evidence-based treatment for individual patients, often through a regular team meeting [4]. Interdisciplinary teams are less formalized structures then MDTs, in which members work between their disciplines to provide a coordinated process of assessment, interpretation, intervention planning and implementation [3,5], rather than from within the boundaries of their own specific disciplines, as is the case for multidisciplinary care [5]. This is particularly pertinent in cancer care where multiple treatments and providers need to be coordinated to enable effective treatment and outcomes [6]. In many countries, MDTs have been recognized as the gold standard for the delivery of care, including in the United Kingdom, where the value of MDTs has been acknowledged at a policy level, and their use is the preferred means of delivering care [7].

MDTs are at the heart of interdisciplinary cancer care [8]. Other forms of interdisciplinary interactions also occur in the sector, including the use of interdisciplinary governance structures to oversee cancer clinics within organizations [9]. Although not specific to cancer care, it has been acknowledged in the literature that cooperation between health professionals has many advantages, including the ability to improve job satisfaction and mental health of members within a team [10] and improve the cost-effectiveness and quality of care delivered to patients. Despite the many advantages of interdisciplinary care, challenges such as interpersonal conflicts between professionals [8] and lack of organizational support [11] need to be overcome to enable effective cooperation among MDTs. The optimal use of technology has been recognized as a key component in overcoming some of the challenges inherent in interdisciplinary health care by improving access to information across care settings and overcoming the logistics of creating linkages between primary and tertiary care providers [12].

Recent literature suggests that integrating ICTs (information communication technology) into MDTs working with brain injury patients has the potential to improve the delivery of care for these patients [13]. Furthermore, research suggests a potential benefit for using ICTs to standardize cancer care and improve the collection of clinical data [14]. In spite of this, research into the use of technology in interdisciplinary cancer care is varied and, at present, there is limited understanding about the breadth and effectiveness of ICT use in multidisciplinary cancer care. This review aimed to identify articles that describe how eHealth facilitates collaboration in interdisciplinary cancer care, and understand the ways in which these eHealth innovations are being used in this setting. In the context of this review eHealth was defined as “the application of information and communications technologies (ICTs) across the whole range of functions that affect health” [15]. This is a broad definition that includes health informatics, health monitoring, telehealth, mobile and app technologies, social media, and online learning.

## 2. Materials and Methods

A systematic search was conducted in March 2015 to identify articles that described how technology facilitates collaboration in interdisciplinary cancer care. To identify more recent publications relevant to the review, the search was repeated using Embase and Medline (via OvidSP) in September 2016. There was no registered protocol for the review.

### 2.1. Inclusion Criteria

The review included articles that reported findings from studies on eHealth and interdisciplinary care and cancer. eHealth was defined as the use of technology to support patient care, education, and research. This included health informatics, health monitoring, telehealth, mobile and app technologies, social media, and online learning. Cancer care was defined as any form of cancer or oncological care. Interdisciplinary was defined as relating to more than one branch of knowledge in care provision, i.e., a team of health professionals working collaboratively was considered interdisciplinary if they were from different disciplines or from the same discipline but had different sub-specialties, such as in medicine. The review included both diagnostic and interventional studies. Additionally, our criteria required that the articles were published in a peer-reviewed journal article, written in English, and only included human subjects.

Articles were excluded from the review if they were written in a language other than English, described robotic surgery, focused on online professional development, or reported on the use of analogue technologies such as the facsimile or telephone.

### 2.2. Search Criteria

A search was conducted in the following electronic databases, academically recognized in the field of health: CINAHL; PubMed; OvidSP (EBM Reviews; Cochrane Database of Systematic Reviews; ERIC; MEDLINE; PsycINFO; The Joanna Briggs Institute EBP Database). Reference lists of articles which met the inclusion criteria were identified and manually searched for further sources. Titles containing any of the search keywords were highlighted and abstracts and/or full papers were searched to assess the eligibility for inclusion. Refer to Table 1 for an example of the search strategy implemented into databases. This entry style was modified as required for use in each database searched.

### 2.3. Study Selection

Search results were exported into the referencing software EndNote ((EndNote X8 (Bld 10063)) for sorting; duplicate and incomplete entries were scanned and removed. For the remaining references, the specified exclusion criteria were applied to remove irrelevant articles based on the title, then title and abstract. The full text was then retrieved and five independent reviewers (authors 1, 2, 3, 5, and 6) assessed eligibility. If the eligibility of the paper was still unclear after reviewing the full text, a consensus decision was made following discussion between the reviewers. Studies that were mutually agreed upon by all reviewers as eligible were selected to be included in the review.

**Figure 1:**
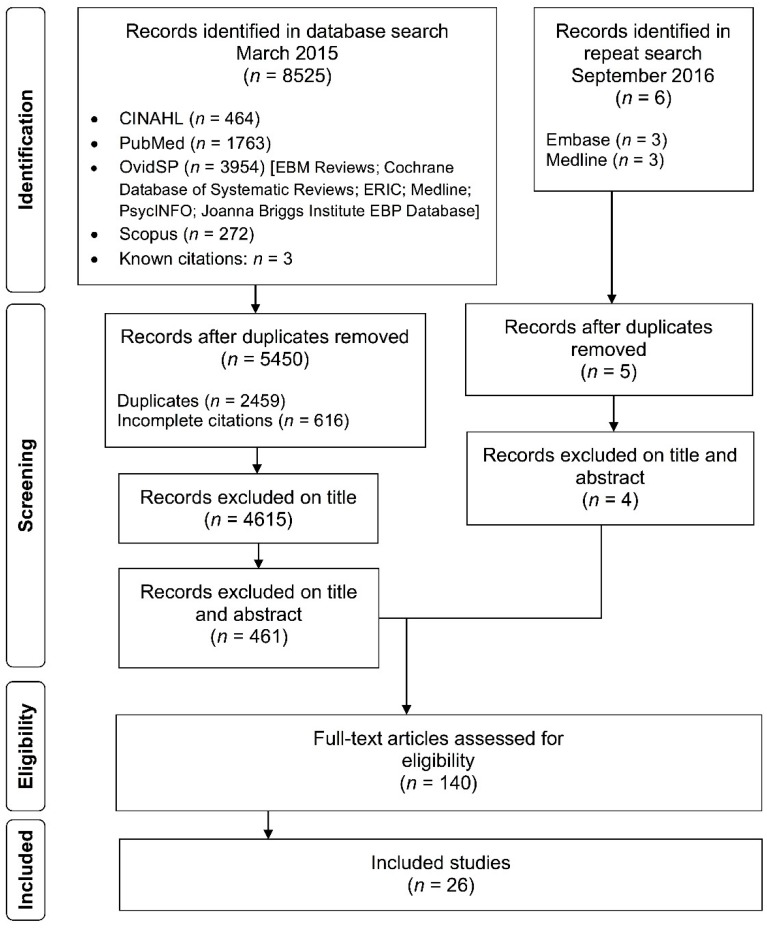
Preferred Reporting Items for Systematic Reviews and Meta-Analyses (PRISMA) [16] flow diagram of the search strategy.

The initial search of the relevant databases returned 8525 citations. Once duplicates (*n* = 2459) and incomplete citations (*n* = 616) were removed, 5450 remained to be reviewed by the research team. An additional 4615 articles were excluded on title alone, and a further 461 were excluded based on title and abstract. A full text review of the remaining 139 articles eliminated an additional 113, leaving 25 articles in the final review. The search was repeated in September 2016, identifying six citations published since the initial search was conducted. Of these, one duplicate was removed, and four were excluded on title and abstract, leaving one additional article for inclusion following full text review, meaning 26 articles were included in the review. Refer to Figure 1 to see the Preferred Reporting Items for Systematic Reviews and Meta-Analyses (PRISMA) [16] flow diagram of the search strategy.

### 2.4. Data Extraction and Analysis

Data was extracted systematically from the included studies by three authors (authors 2, 5, and 6). The following information was extracted from each article included in the review: (i) citation (including country); (ii) eHealth component (i.e., ICT applied specifically to support health and well-being); (iii) interdisciplinary component (i.e., the collaborative process supported by the ICT); (iv) cancer type; (v) setting; and (vii) study design. No standardized tools were used to conduct a formal assessment of the quality of the studies. Results for this review are presented as a qualitative synthesis of descriptive data. An integrative analysis of findings was conducted, where individual categories in the results of each study were identified and coded to identify themes [17] that emerged across the studies.

## 3. Results

An analysis of the data extracted from the 26 included articles [7,18,19,20,21,22,23,24,25,26,27,28,29,30,31,32,33,34,35,36,37,38,39,40,41,42] revealed five broad themes:

1. Data collection and accessibility (*n* = 2)
2. Virtual multidisciplinary teams (*n* = 12)
3. Communication between individuals involved in the delivery of health services (*n* = 5)
4. Communication pathways between patients and cancer care teams (*n* = 4)
5. Health professional-led change (*n* = 3)

Refer to Table 2 to see an overview of the articles, the theme that they align with, the eHealth component of the study, and the multidisciplinary component of the study.

### 3.1. Data Collection and Accessibility

This review identified two studies (8%) reporting findings on the use of technology to improve data collection for, and feedback to, individuals involved in the delivery of interdisciplinary cancer care [35,39]. Findings from this theme show that involving a multidisciplinary group in the design of a clinical database results in modification to the database to align better with the needs of the health professionals using it [35]. Changes made to the database as a result of multidisciplinary feedback were anticipated to result in improved quality of services and reporting targets, reducing the cost and time required to deliver care, and improved communication between the health care team, but outcomes of this process are yet to be formally evaluated. The use of technology to feedback patient history data to MDTs was shown to enable more efficient clinical discussion during team meetings and potentially improve interactions with patients [39]. Furthermore, use of technology for feedback of clinical data offered educational advantages to both medical and non-medical team members, including allowing participants to learn anatomy, providing information on disease manifestations and histopathology.

### 3.2. Virtual Multidisciplinary Teams

This theme was the most common one in the review. A total of twelve articles (46%) presented findings on the concept of virtual MDTs, i.e., MDTs that used video or teleconferencing technologies to support meetings [7,22,23,27,28,29,31,32,36,38,40,41]. The articles covered the use of video and teleconferencing technology to support MDT meetings across a wide range of tumour streams: breast [7,22,23,27,28,32], lung [41], urological and gastrointestinal [7], and head and neck [38]. There were consistent advantages and disadvantages identified across the articles in regard to using video and teleconferencing technology to connect MDT members during meetings. The advantages of using videoconferencing technology to connect MDT members during meetings included the ability to include teams with dispersed members, the potential reduced treatment time for patients and improved coordination of care [23,31], and the ability to connect primary care providers with specialists [38,40,41]. The disadvantages of using video and teleconferencing to connect MDTs included concerns about privacy and confidentiality of patient data [38], the need for additional preparation for MDT meetings [32], challenges in coordinating meeting times [38] and unreliability of technology [22,36].

A number of articles also focused on aspects of using video and teleconferencing technology to connect MDTs beyond the barriers and enablers. Aspects of using video and teleconferencing technologies to connect MDTs discussed in the articles included factors affecting ICT adoption in MDTs, the cost effectiveness of implementing ICT, and comparisons between technology supported and face-to-face only MDTs. MDT members with less video conferencing experience, or who were from allied health disciplines were generally less supportive of tele and video conferencing in MDTs [28]. Cost concerns from the implementation and adoption of virtual MDTs included a need for reimbursement for participation in virtual teams, as opposed to face-to-face teams [38], and delay in financial returns from implementing virtual MDTs [28]. In comparison with face-to-face MDTs, patients supported by virtual MDTs had improved access to specialized care [41] and health professionals reported valuable discussions with colleagues from different specialties [38], timelier specialist input, and greater access to professional development [41].

### 3.3. Communication between Individuals Involved in the Delivery of Health Services

Five articles (19%) examined the use of technology to support interdisciplinary communication in cancer care, outside of the context of an MDTM [20,26,30,37,42]. ICT could complement (but not replace) current practices for supporting interaction between health professionals involved in the delivery of cancer care to facilitate early detection of patient problems [37]. The use of eHealth in multidisciplinary cancer care was also shown to improve the delivery of care to patients by increasing service capacity [42]. Furthermore, it was identified that both primary and tertiary health professionals involved in the delivery of cancer care were supportive of the use of technologies to improve their interaction pathways [30]. However, barriers to using technology to support communication between individuals involved in the delivery of cancer services were identified. Barriers included difficulties using technology to support interaction with health care providers outside a single organizations cancer care clinic due to technological barriers such as firewalls [30] and reticence or lack of time for using communication systems, coupled with technical problems [20]. Although barriers to the use of technology to support professional communication were identified, it was also concluded that investing in implementation strategies that considered environmental and personnel factors could overcome barriers [20].

One article presented findings of a very different eHealth intervention to connect local clinicians with specialists in remote locations. This intervention consisted of the use of a smartphone worn by the patient as an in vivo sensor network to monitor those undergoing radiotherapy and chemotherapy [37]. The smartphone established collaboration between local clinicians and remote experts of different treatment modalities. This involved the local clinician inputting details about the cancer type and position into the device, which then connected to remote experts in hyperthermia, radiotherapy, and chemotherapy. Remote experts provided guidance to the treating clinicians based on the data from the smartphone; this also updated them on treatment outcome.

### 3.4. Communication Pathways between Patients and Cancer Care Teams

The review of the literature identified four articles (15%) that examined the use of technology to support interdisciplinary communication in cancer care between patients and cancer care teams [18,19,24,25]. All the articles stated that telemedicine improved access to specialist health services for cancer patients. A number of the articles presented additional findings.

Three articles presented results on the use of eHealth to collect patient-reported data for feedback to the interdisciplinary cancer team. The findings showed that administrative staff concerns about ICT impacts on workload presented a key barrier to implementing eHealth in cancer centers [19]. On the other hand, clinicians reported that eHealth innovations enhanced monitoring of patients so as to improve treatment outcomes [19] and patient self-efficacy regarding their condition [25]. Patients reported high satisfaction with eHealth tools for connecting them with their cancer MDTs [25] and for enhancing their understanding of their condition [24]. Consistent across these three papers, patients indicated that they would recommend various eHealth services/tools to other patients undergoing treatment.

The final article was a case study evaluating the information systems personnel and processes involved in an eHealth service for providing mobile mammography to patients [18]. The authors identified a number of benefits of mobile mammography including the ability for patients to receive a mammogram the day they visit a community health care provider, reduced wait time for the patient, and timely follow up by the healthcare team. The challenges to implementing mobile mammography include technological difficulties, ill-defined organizational goals, and differences across organizational communication and information systems. However, the article indicates challenges can be overcome by investing in a dedicated specialist team to deliver mobile mammography, addressing technology connectivity issues, enabling patient pre-registration for the service and educating host sites and patients about what to expect on their day of visit.

### 3.5. Health Professional-Led Change

A total of three articles (12%) analyzed in this review focused on the role of health professionals in driving change regarding eHealth implementation [21,33,34]. Two of the articles presented findings on the barriers and enablers of eHealth implementation in interdisciplinary cancer care [21,33]. The third article discussed findings about the technical literacy of health professionals involved in interdisciplinary cancer care [34].

Evans et al. [21] identified the following as strategies for overcoming barriers to implementing a clinical information system across multiple healthcare organizations: organizational acceptance of the need for change, engaging leaders in fostering a culture of change, anticipating and planning for technology implementation and interface related barriers, and providing resources and support during implementation, including education and training for staff in using new systems. Some of these principles were applied in the implementation of an electronic patient record system within a cancer clinic [33], specifically the planning for technology and resource barriers by installing computer stations and hand-held tablet computers so data could be input in real time. The result of this implementation strategy was not clearly articulated in the article, but it was noted that the success of the eHealth intervention for data collection varied for different individuals in the clinic. The final article in this theme [34] presented the results of a survey of 55 clinicians working in cancer care, concluding that there is a need to adapt mainstream technologies for specific institutional settings particularly in regard to making it useful for individual patient needs.

## 4. Discussion

The aim of this review was to identify and describe the peer-reviewed literature on how eHealth facilitates collaboration in interdisciplinary cancer care. Analysis of these 26 articles revealed considerable diversity of eHealth use across cancer care, but low usage of the term eHealth to present findings relating to its use in the literature. The inconsistency in the literature on the use of eHealth to describe technology use in cancer care may reflect a broader challenge facing the health sector around a consistent definition of eHealth. The lack of consistent terminology describing eHealth has been recognized in the literature [43,44], and identified as a potential barrier to the adoption of consistent eHealth practices by health professionals.

Additionally, this review indicated that much of the research into eHealth in interdisciplinary cancer care uses a qualitative methodology [21,24,34], or does not clearly describe the methodology used. This is likely because a number of articles described the process of developing and implementing eHealth in specific organizations [18,33], rather than evaluating the outcomes of the technologies.

This review highlights the breadth of eHealth use among cancer MDTs. It is clear that technology is being used in areas such as MDT communication [7,22,23,27,28,29,31,32,36,38,40,41], communication between professionals delivering cancer care [20,26,30,37,42] and for supporting patient communication with their healthcare team [18,19,24,25]. Notably, there was a predominant focus on multidisciplinary care rather than interdisciplinary eHealth practice, which is consistent with recent findings in other fields of eHealth research [45,46]. Additionally, there are still a number of areas of interdisciplinary cancer care where the use of eHealth is either less widely utilized, or less widely researched. A particularly notable area that is under-researched is how technology is supporting interdisciplinary teams to become drivers of change, with only three articles presenting findings relating to this theme [21,33,34]. There are also a number of areas where there appears to be no literature on the use of eHealth in cancer care, including the use of online interventions to enhance clinical communication or clinician researcher communication and research into the use of smartphone apps in interdisciplinary cancer care.

Although it has been acknowledged that technology has the potential to be of value in interdisciplinary care, findings from this review suggest there is little research focused on how eHealth can be implemented effectively in interdisciplinary cancer care. The most researched area was the use of video and teleconferencing technology to support virtual MDT meetings [7,22,23,27,28,29,31,32,36,38,40,41]. In the context of virtual MDTs, technology is used to connect teams where specialists are geographically dispersed and, in one instance, technology was used to create linkages between primary and tertiary care facilities [38]. Findings from this literature identified a number of barriers to the use of eHealth to connect MDTs including reliability of technology, and an increased burden on workloads. These findings are similar to the wider telehealth and social media literature, which suggests that health professionals are supportive of eHealth for patient and health professional communication, but resistant to integrating it directly into service provision [47]. In spite of these barriers, almost all the literature acknowledges benefits of the use of technology to support virtual MDTs for both patients and health professionals [36,38,40].

A small number of articles presented findings on the use of end user feedback or co-design in the design and implementation of eHealth interventions in cancer care. Articles that did use co-design identified benefits of involving interdisciplinary cancer care professionals in the design of an eHealth innovation [7,33]. Furthermore, it was interesting how few articles specifically identified technologists, such as computer scientists, data analysts or other experts in the development, design and evaluation of technology as being involved in the co-design process for eHealth applications. In this review, only one article identified technologists as being involved in the design of an eHealth intervention [19].

Finally, this review revealed that research into eHealth applications in interdisciplinary cancer care is much more focused on development and implementation on interventions than on long term impact. Few articles reported systematic evaluations of the effectiveness of (a) the implementation process, and (b) the intervention on treatment quality, MDT processes, and patient outcomes. The notable exception to this is in telemedicine, where some articles described the long term impact of eHealth on both patients and cancer services [37]. This may be due to the fact that this area of eHealth is well developed and has existed for several decades [48], whereas the use of smartphones in healthcare is less than a decade old, due to the technology only being available since the mid-2000s.

## 5. Conclusions

Technology is having a transformative effect on the health sector. In the area of cancer care, its use is widespread, particularly in supporting interdisciplinary collaboration in MDTs and to connect patients with specialist care teams. However, notable gaps remain in the use of eHealth, such as smartphone apps and online resources for research and education in interdisciplinary cancer care. It is imperative that further research is undertaken on the design, evaluation and long-term sustainability of eHealth in interdisciplinary cancer care. Technology evolves quickly and it is the responsibility of researchers to provide health professionals guidance on how best to respond to the impact of technology in the health sector.

## Figures and Tables

**Table 1:** Example of the search strategy implemented into databases.

Step	Key Words
1	(eHealth) apps OR digital OR eHealth OR elearning OR electronic health record * OR emedicine OR ePrescribing OR etherap * OR health technolog * OR ICT OR instant messag * OR information technolog * OR internet OR mhealth OR MMS OR mobile * OR online OR podcast * OR smartphone * OR SMS OR social media OR technolog * OR telecare OR telehealth OR telemedicine OR telerehabilitation OR telecommunication * OR teleconference * OR videoconference * OR virtual OR web * based OR website *
2	(interdisciplinary) interdisciplinarity OR interdisciplinary OR collaborat * OR mulitdisciplinary OR team * OR transdisciplinary OR interprofessional
3	1 and 2
4	(cancer) blastoma OR cancer * OR carcinoma * OR leukaemia OR leukemia OR lymphoma OR lymphedema OR malignant OR melanoma OR mesothelioma OR metast * OR neoplas * OR pseudomixoma OR sarcoma OR tumo *
5	3 and 4

* Symbol denotes search has been boarded by finding words that start with the same letters, but have different endings.

**Table 2:** Overview of the articles, themes, eHealth components, and interdisciplinary components of the included studies articles.

Reference	Methodology	eHealth Component	Multidisciplinary Component
*Theme One: Data collection and accessibility*			
Raptis, D.A. et al., 2011 [35]	Qualitative	Developed web-based software on a Microsoft 2000 SQL Server database. The software design is available as open access and can be requested for free from the authors for implementation in any national health system worldwide.	Engaged with junior and senior doctors, specialist nurses, endoscopy unit, imaging department, the clinical risk management committee, and patients, in development of this system.
Simo, R.P. et al., 2009 [39]	Other	Integrated media presentation (Microsoft PowerPoint) presented in multidisciplinary team meetings ^1^.	Multidisciplinary head and neck meeting.
*Theme Two: Virtual Multidisciplinary Teams*			
Fielding, R.M. et al., 2005 [22]	Qualitative	Using videoconferencing technology to conduct multidisciplinary team meetings with health professionals at different sites.	Connected breast multidisciplinary team ^2^ members.
Fitzpatrick, D.D. et al., 2012 [23]	Qualitative	Using videoconferencing technology to connect health professionals in order to conduct virtual consultations (VCP)s.	Connected radiation oncology and spinal surgery for the management of patients diagnosed with spinal cord compression.
Jalil, R.M. et al., 2013 [7]	Qualitative	Using videoconferencing technology in multidisciplinary team meetings.	Participants included urologists, gastrointestinal surgeons, oncologists, cancer nurses, radiologists and histopathologists.
Kunkler, I.H. et al., 2007 [28]	Cluster randomized controlled trial	Using videoconferencing technology to conduct multidisciplinary team meetings with health professionals at different sites.	Connected breast MDT: surgeons, radiologists, pathologists, oncologists, breast care nurses.
Kunkler, I.H. et al., 2005 [27]	Mixed	Using videoconferencing technology to conduct multidisciplinary team meetings with health professionals at different sites.	Connected breast multidisciplinary team: surgeons, radiologists, pathologists, oncologists, breast care nurses.
Li, J. and Robertson, T. 2011 [29]	Qualitative	Using videoconferencing technology in multidisciplinary team meetings.	Connected multidisciplinary team: surgeons, radiologists, pathologists, medical oncologists, radiation oncologists, psychologists, oncology nurses and social worker.
Murad, M. et al., 2014 [31]	Other	Using videoconferencing technology in multidisciplinary team meetings.	Connected multidisciplinary teams: surgeons, radiologists, histopathologists and oncologists.
Murthy, V. et al., 2014 [32]	Mixed	Using videoconferencing technology in multidisciplinary team meetings	Connected breast multidisciplinary teams: breast surgeons, plastic surgeons, radiation and medical oncologists, radiologists, pathologists, residents, medical students and nurses.
Robertson, T. et al., 2010 [36]	Qualitative	Using videoconferencing technology in multidisciplinary team meetings.	Connected breast multidisciplinary team members.
Shea, C.M. et al., 2014 [38]	Qualitative	Using videoconferencing technology in Multidisciplinary team meetings.	Connected multidisciplinary team members.
Stalfors, J. et al., 2005 [40]	Other	Using videoconferencing technology in Multidisciplinary team meetings	Multidisciplinary team members included specialists in oncology, radiology and pathology. When appropriate, specialists in dental surgery, oral surgery, general surgery, thoracic surgery, dietetics and other specialists are engaged. Patients attend unless too unwell or follow-up case presentation.
Stevens, G.J. et al., 2012 [41]	Other	Using videoconferencing technology to conduct Multidisciplinary team meetings with health professionals at different sites.	Connected multidisciplinary team members: respiratory physicians, thoracic surgeons, radiation oncologists, medical oncologists and a diagnostic radiologist.
*Theme Three: Communication between individuals involved in the delivery of health services*			
DuBenske, L.L. et al., 2010 [20]	Other	An online interactive health communication system (IHCS) to bridge the communication gaps that occur among patients, family, and clinicians and to empower each to actively engage in cancer care and shared decision making. IHCS can facilitate discussions amongst multiple disciplines within a single forum.	Engaged doctors, patients, carers, other relevant health professionals.
Kamal, R.C. et al., 2012 [26]	Other	A smartphone on the body that controls an in vivo sensor network deployed for use in hyperthermia, radiotherapy, and chemotherapy. The smartphone schedules temperature using an algorithm and provides subscriber, publisher, broker role, and cluster information to all in vivo nodes, and receives subscription or notification confirmation from other in vivo nodesvia Bluetooth or Zigbee technology using an area network. The smartphone updates the electronic health record (EHR ^3^)/patient health record (PHR), prior to and after treatment. The smartphone application was implemented in Android SDK version 4.0 and the doctor’s panel for the CTCU with a web interface.	Enables communication between local clinicians and remote experts of different treatment modalities. Local clinician inputs cancer type and position in the local cancer treatment control unit (CTCU), and a hypervisor ascertains experts on hyperthermia, radiotherapy, and chemotherapy. It then communicates with the remote CTCU to connect to remote experts through a smartphone. Remote experts recommend drug measurement, radiation measurement, and heating temperature to the smartphone kept near the patient.
Morton, C.A. et al., 2011 [30]	Qualitative	Photo-triage for suspected skin cancers. High-quality close-up dermascopic images are taken in primary care location. These are then sent to specialist for review, so patients can be triaged in to specific treatment clinics.	Connected primary care providers and dermatology specialist.
Sada, Y.H. et al., 2011 [37]	Qualitative	Three integrated health systems that used electronic health records (EHRs).	Connected patients, oncologists and other physicians.
Van den Brink, J. et al., 2005 [42]	Qualitative	Electronic health information support system.	Connected head and neck cancer patients, hospital physicians, members of a hospital-based support team, GPs, district nurses and speech therapists.
*Theme Four: Communication pathways between patients and cancer care teams*			
Browder, C.J. et al., 2015 [18]	Other	Case study evaluating the information systems, personnel, and processes involved in mobile mammography settings.	Involved interviews with participants from a hospital involved in mammography included nurses and radiologists.
Clark, K.W. et al., 2009 [19]	Qualitative	Technology to support real-time communication between patients and health care team regarding psychosocial problem-related distress. Uses touch-screen technology.	Involved Information Communication Technology experts, nurses, doctors, social workers, statisticians in development of the program.
Gordon, J. and Gruber, M. 2012 [24]	Qualitative	Use of virtual visits by health professionals to care for patients attending chemo infusion centers. Technology used for tele-dermatology to maintain the continuity of the patients’ care when moving between primary and tertiary care.	Involved multidisciplinary team comprising of clinical, administrative, planning, and other representatives charged to identify and develop a location that would provide oncology care for patient’s closer to home and improve the patients’ experience.
Head, B.A. et al., 2009 [25]	Other	A telehealth intervention to address isolation, develop patient self-efficacy, and improve symptom management during the treatment experience. Health Buddy^®^, a product of the Health Hero Network (Palo Alto, CA, USA), was the appliance chosen to communicate the intervention algorithms.	Involved multidisciplinary team members: surgical, medical, and radiation oncologists as well as representatives from nursing, social work, psychology, speech therapy, and nutrition therapy.
*Theme Five: Health professional led change*			
Evans, W.K. et al., 2014 [21]	Qualitative	Development of a regional oncology information system for sharing information and patient data.	Involved consultation with various health professionals and workplace managers and executives.
Nwagwu, W.E. et al., 2013 [33]	Other	Case study exploring the way of Information Communication Technology in cancer care facilitate health information sharing. Included looking at Information Communication Technologies at point of care, electronic health records, clinical decision support tools, and order entry systems.	A cancer care group made up of health professionals from relevant disciplines such as nurses, physiotherapists, health psychologists, physicians, occupational therapists, lay public, lawyers and pharmacists among others, all serving as volunteers.
Oborn, E.M. et al., 2011 [34]	Qualitative	A web-based clinical information system (SubSys) was implemented. SubSys was used to record cancer-related information pertaining to patients seen in the clinics.	Engaged multidisciplinary team: surgeons, oncologists, pathologists, radiologists, and nurse specialists

^1^ MDTM—Multidisciplinary Team Meeting; ^2^ MDT—Multidisciplinary Team; ^3^ HER—Electronic Health Record.
